# Experimental infection with a Thai reassortant swine influenza virus of pandemic H1N1 origin induced disease

**DOI:** 10.1186/1743-422X-10-88

**Published:** 2013-03-16

**Authors:** Nataya Charoenvisal, Juthatip Keawcharoen, Donruethai Sreta, Siriporn Tantawet, Suphattra Jittimanee, Jirapat Arunorat, Alongkorn Amonsin, Roongroje Thanawongnuwech

**Affiliations:** 1 Department of Pathology, Faculty of Veterinary Science, Chulalongkorn University, Henri-Dunant Rd, Bangkok 10330, Thailand; 2 Department of Veterinary Microbiology, Faculty of Veterinary Science, Chulalongkorn University, Henri-Dunant Rd, Bangkok 10330, Thailand; 3 Faculty of Veterinary Medicine, Rajamangala University of Technology Tawan-ok, Bangpra, Chonburi 20110, Thailand; 4 Department of Clinical Science and Public Health, Faculty of Veterinary Science, Mahidol University, Salaya, Nakhon Pathom 73170, Thailand; 5 Department of Veterinary Public Health, Faculty of Veterinary Science, Chulalongkorn University, Henri-Dunant Rd, Bangkok 10330, Thailand; 6 Emerging and re-emerging infectious diseases in animals, Research unit, Faculty of Veterinary Science, Chulalongkorn University, Henri-Dunant Rd, Bangkok 10330, Thailand

**Keywords:** Influenza, Pandemic H1N1 2009, Pathogenesis, Reassortant, Swine, Thailand

## Abstract

**Background:**

Following the emergence of the pandemic H1N1 influenza A virus in 2009 in humans, this novel virus spread into the swine population. Pigs represent a potential host for this virus and can serve as a mixing vessel for genetic mutations of the influenza virus. Reassortant viruses eventually emerged from the 2009 pandemic and were reported in swine populations worldwide including Thailand. As a result of the discovery of this emergent disease, pathogenesis studies of this novel virus were conducted in order that future disease protection and control measures in swine and human populations could be enacted.

**Methods:**

The pandemic H1N1 2009 virus (pH1N1) and its reassortant virus (rH1N1) isolated from pigs in Thailand were inoculated into 2 separate cohorts of 9, 3-week-old pigs. Cohorts were consisted of one group experimentally infected with pH1N1 and one group with rH1N1. A negative control group consisting of 3 pigs was also included. Clinical signs, viral shedding and pathological lesions were investigated and compared. Later, 3 pigs from viral inoculated groups and 1 pig from the control group were necropsied at 2, 4, and 12 days post inoculation (DPI).

**Results:**

The results indicated that pigs infected with both viruses demonstrated typical flu-like clinical signs and histopathological lesions of varying severity. Influenza infected-pigs of both groups had mild to moderate pulmonary signs on 1-4 DPI. Interestingly, pigs in both groups demonstrated viral RNA detection in the nasal swabs until the end of the experiment (12 DPI).

**Conclusion:**

The present study demonstrated that both the pH1N1 and rH1N1 influenza viruses, isolated from naturally infected pigs, induced acute respiratory disease in experimentally inoculated nursery pigs. Although animals in the rH1N1-infected cohort demonstrated more severe clinical signs, had higher numbers of pigs shedding the virus, were noted to have increased histopathological severity of lung lesions and increased viral antigen in lung tissue, the findings were not statistically significant in comparison with the pH1N1-infected group. Interestingly, viral genetic material of both viruses could be detected from the nasal swabs until the end of the experiment. Similar to other swine influenza viruses, the clinical signs and pathological lesions in both rH1N1 and pH1N1 were limited to the respiratory tract.

## Background

Influenza A viruses are highly contagious respiratory pathogens capable of transmission between various avian and mammalian species including swine and humans. Two specific receptors: sialic acid (SA) α2,3 commonly found in the epithelial cells of gastrointestinal tract of wild aquatic birds and SA α2,6 found in the epithelial cells of the respiratory tract of humans, are recognized. Pigs are known as a “mixing vessel” as they express receptors which can bind both avian and human influenza viruses within the respiratory tract. As a result, interspecies transmission from pigs to humans or vice versa is possible. A study of pig-to-human influenza virus transmission on Thai swine farms proved that swine-exposed workers had antibodies against the circulating swine influenza viruses (SIV) [1]. Cross-species transmission becomes an important factor in monitoring for future human influenza outbreaks. Pandemic H1N1 (pH1N1) virus emerged in April 2009 and rapidly spread among human populations globally. The pH1N1 virus was also called Swine-origin 2009 A (H1N1) due to all of its gene segments closely related to SIV. The pH1N1 virus is a reassortant virus of the European avian-like swine virus (M and NA genes), the classic swine H1N1 virus (HA gene) and the North American triple reassortant H3N2 virus (PB2, PB1, PA, NP and NS genes) [2]. Following the epidemic outbreaks within the human population, the virus was also isolated from pigs in Canada, Norway, Italy, Hong Kong, South Korea and Thailand [3-8]. It should be noted that the North American triple reassortant internal gene (TRIG) virus might influence antigenic drift and shift in mammalian species [9]. As a result, the reassortant variants of pH1N1 containing TRIG cassette were occasionally found in swine and other animals including turkeys [8,10-15]. The recent Thai reassortant pH1N1 (rH1N1) virus has 7 genes derived from the pH1N1 virus and has only the Neuraminidase (NA) gene from an endemic Thai swine H1N1 virus [12]. Thus, amino acid sequences of Hemagglutinin (HA) gene of the pH1N1 and rH1N1 are 98.4% identical and most antigenic sites are quite similar.

Previous pathological studies comparing the pH1N1human isolate and a seasonal human H1N1 influenza virus in pigs found that those pigs showed none of the clinical signs associated with SIV [16]. Microscopic lesions revealed only mild bronchitis and bronchiolitis with peribronchiolar lymphocytic cuffing and a mild interstitial pneumonia [17]. The pathology of the virus having undergone reassortant in pigs demonstrated in the present experiment may reflect severity of disease not only in pigs but also in humans. In addition, individuals working closely with infected swine may facilitate a human-animal interface, thereby promoting viral transmission between humans and pigs [1]. Interestingly, the genetics of SIV circulating in North America in 1997-1998 were not considered to be stable when the triple reassortant H3N2 virus was introduced resulting insignificant febrile disease, severe influenza-like illness, mortality in piglets and abortion in sows. As a result, surveillance and pathogenesis studies are considered to be essential due to this highly evolved genetic variation of SIV in North America [18,19].

In the present experiment, a pathogenesis study of pH1N1 and its reassortant pH1N1 (rH1N1) following experimental infection of three week old piglets has demonstrated that acute respiratory disease in nursery pigs is induced by both viruses. Pigs in the rH1N1-infected group showed prominent clinical signs, with higher numbers of animals shedding the virus, increased severity of pulmonary lesions and evidence of viral antigen in lung tissue. The information gained from the present study confirmed the increased virulence of the reassortant influenza virus in comparison with the pandemic virus.

## Results

### Clinical examination

Clinical signs were noted daily at 1-7, 10 and 12 days post infection (DPI) in both cohorts. One pig from the pH1N1-infected group (group 1) was found dead due to stress following restraint and findings associated with this animal were excluded from our evaluation. The pH1N1-infected pigs developed sneezing (3 of 8) and had ocular discharge (1 of 8) beginning at 1-2 DPI, and subsequently showed mild (2 of 8) to moderate (3 of 8) serous nasal discharge and conjunctivitis (5 of 8) at 2 DPI. In contrast, the rH1N1-infected pigs showed increased severity of clinical signs, with moderate to severe serous nasal discharge (8 of 9), sneezing (5 of 9) and conjunctivitis (9 of 9) at 1-2 DPI with resolution of the former two clinical signs and amelioration of the discharge at 3-4 DPI in 5 of 6 animals. Only mild serous nasal discharge was observed in two pigs in both cohorts at the end of the experiment (12 DPI). Pigs in the control group had no signs of disease throughout the course of the experiment.

### Viral shedding

Viral shedding was measured from nasal swabs using a modified real time RT-PCR and viral isolation in MDCK cells (Table 1). One pH1N1-infected pig (1 of 8) demonstrated evidence of viral shedding as early as 1 DPI. One of the six remaining pigs in the same group was tested positive at 2 DPI with very low levels of viral copies (data not shown). Subsequently, one of the two remaining pigs was tested positive at 5 DPI by both real time RT-PCR and viral isolation. By day 7 post infection, all of the pH1N1-infected pigs shed the virus with high levels of viral copies at 7, 10 and 12 DPI from collected nasal swabs (data not shown). In the rH1N1-infected cohort, one pig was tested positive as early as 1 DPI followed by five of nine animals being positive at 2 DPI, while all six remaining pigs were tested positive at 3 DPI by both real time RT-PCR and viral isolation tests. Similar to the pH1N1 group, viral shedding in nasal swabs was detected again at 10 DPI (2 of 3 pigs) and was detected in all the remaining pigs (3 of 3) at 12 DPI by the real time RT-PCR. None of the nasal swabs from the control group yielded positive results from both tests.

**Table 1:** Viral shedding measured from nasal swabs detected by a real time RT-PCR and viral isolation

**Animal ID**	**Virus detection**																			
	** 0 DPI**		** 1 DPI**		** 2 DPI**		** 3 DPI**		** 4 DPI**		** 5 DPI**		** 6 DPI**		** 7 DPI**		** 10 DPI**		** 12 DPI**	
**pH1N1-inefected group**	rt	VI	rt	VI	rt	VI	rt	VI	rt	VI	rt	VI	rt	VI	rt	VI	rt	VI	rt	VI
1	-	-	-	-	-	-	N													
2	-	-	-	-	-	-	N													
3	-	-	-	-	-	-	-	-	-	-	N									
4	-	-	-	-	-	-	-	-	-	-	+	+	-	+	+	-	+	-	+	-
5	-	-	+	-	-	-	-	-	-	-	-	-	-	-	+	-	+	-	+	-
6	-	-	-	-	-	-	-	-	-	-	N									
7	-	-	-	-	+	-	-	-	-	-	N									
8	-	-	-	-	-	-	N													
**rH1N1-infected group**																				
1	-	-	-	-	+	-	+	+	-	-	N									
2	-	-	-	-	-	-	+	-	-	-	-	-	-	-	-	-	-	-	+	-
3	-	-	-	-	+	-	+	+	-	-	-	+	-	-	-	-	+	-	+	-
4	-	-	-	-	-	-	+	-	-	-	N									
5	-	-	-	-	-	-	+	-	-	-	-	-	-	+	-	-	+	-	+	-
6	-	-	-	-	-	-	N													
7	-	-	-	-	+	-	N													
8	-	-	+	-	+	-	N													
9	-	-	-	-	+	-	+	+	-	-	N									
**Negative control group**																				
1	-	-	-	-	-	-	-	-	-	-	-	-	-	-	-	-	-	-	-	-
2	-	-	-	-	-	-	-	-	-	-	N									
3	-	-	-	-	-	-	N													

*DPI* Day post infection.

*rt* A real-time RT-PCR (+ = Ct values < 40; - = Ct values ≥ 40).

*VI* Viral isolation using MDCK cell line.

*N* Necropsy.

Subsequent to staggered endpoints within the study, euthanasia and necropsy, viral detection in bronchial lymph node and lung tissues was performed (Table 2). In the pH1N1-infected group, virus was detected in 1 lung sample and 2 bronchial lymph nodes when necropsied at 2 DPI by a real time RT-PCR but virus isolation yielded negative results. In contrast, the virus was detected in all rH1N1-infected lungs at 2 DPI and one lung sample (1 of 3) at 4 and 12 DPI by both real time RT-PCR and virus isolation tests. The virus genetic material was also detected in bronchial lymph node of two rH1N1-infected pigs (2 of 3) at 2 and 4 DPI by the real time RT-PCR. The viral genetic material could not be detected in the sera of all pigs analyzed. All control pigs were negative for influenza virus by both tests throughout the experiment.

**Table 2:** Percentages of gross lung lesions and virus detection in lungs and bronchial lymph nodes

**Necropsy day**	**Animal ID**	**Lung lesion (%)**	**Viral detection in lung**		**Viral detection in bronchial lymph node**	
	**pH1N1-inefected group**		**rt**	**VI**	**rt**	**VI**
**2 DPI**	1	0	-	-	-	-
	2	8	-	-	+	-
	9	10	-	-	+	-
**4 DPI**	3	5	-	-	-	-
	7	0	-	-	-	-
	8	5	-	-	-	-
**12 DPI**	4	0	-	-	-	-
	5	0	-	-	-	-
	**rH1N1-infected group**					
**2 DPI**	6	18	+	+	-	-
	7	13	+	+	+	-
	8	13	+	+	+	+
**4 DPI**	1	9	+	+	+	-
	4	0	-	-	-	-
	9	0	-	-	+	-
**12 DPI**	2	0	+	-	-	-
	3	0	-	-	-	-
	5	0	-	-	-	-
	**Negative control group**					
**2 DPI**	3	0	-	-	-	-
**4 DPI**	2	0	-	-	-	-
**12 DPI**	1	0	-	-	-	-

*DPI* Day post inoculation.

*r*t A real time RT-PCR (+ = Ct values < 40; - = Ct values ≥ 40).

*VI* Viral isolation using MDCK cell line.

### Pathological examination

**Figure 1:**
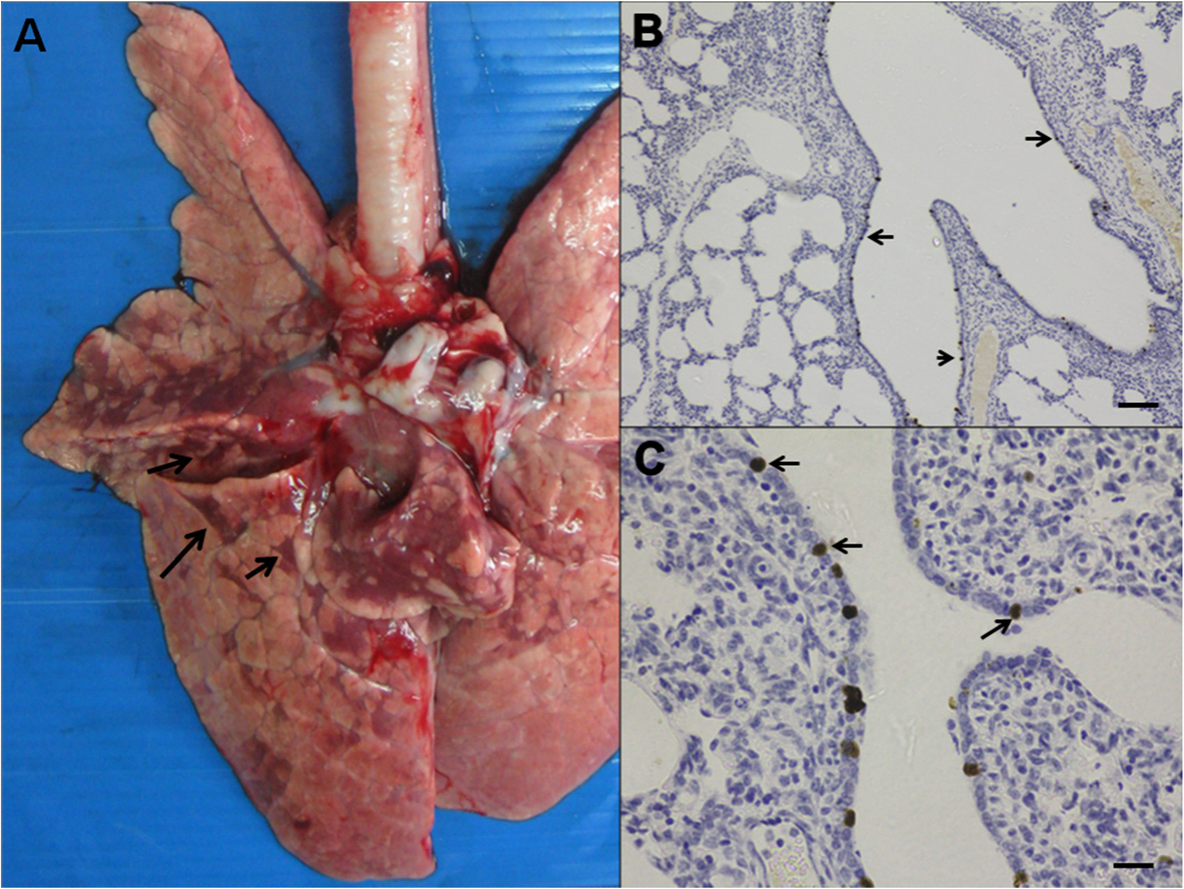
**Gross lung lesion scoring (18%) demonstrated dark plum-color, multifocal to coalescing consolidation or “checker board lung pattern” (arrow) of the rH1N1-infected pig at 2 DPI (A).** Histologically, dark brown staining of the influenza nucleoprotein demonstrating by IHC were observed in the nuclei of the infected bronchial epithelail cells (arrow) (**B**; bar = 200 μm, **C**; bar = 20 μm) from the same rH1N1-infected pig at 2 DPI.

Typical SIV macroscopic lung lesions are characterized by multifocal, dark, plum-colored lungs suggestive of consolidation. In our experimental animals, pH1N1-infected pigs at 2 DPI (1 of 2), 4 DPI (2 of 3) and in all rH1N1-infected pigs at 2 (3 of 3) and 4 (3 of 3) DPI had similar lesions concentrated within the cranioventral regions of multiple lobes (Figure 1A). The lung lesions mentioned above were not related to *Mycoplasma hyopneumoniae* (M. hyo)-induced lesion since the PCR tested for M. hyo detection yielded negative results (data not shown). The percentages of gross lung lesions at 2 DPI demonstrated that the rH1N1-infected pigs had greater lung scores than those of the pH1N1-infected pig (Table 2). Other non-specific gross lesions in both infected groups included mild bronchial lymph node enlargement with multifocal subcapsular hemorrhage.

Microscopic pulmonary lesions were noted as a mild to moderate broncho-interstitial pneumonia in all infected pigs of both groups particularly at 2 and 4 DPI. However, immunohistochemistry (IHC) staining only demonstrated the SIV nucleoprotein in the nuclei of bronchial epithelial cells of all rH1N1-infected pigs at 2 and 4 DPI (Figure 1B and 1C) while none of the pH1N1-infected pigs were found to be immunoreactive. It should be noted that the lesions were limited only in the lungs of the infected pigs and not to any of the other examined tissues. No significant histopathological findings or IHC immunoreactivity were found in any of the control animals.

### Hemagglutinination inhibition (HI) assay

All sera from pre-experiment and terminal blood draws showed negative results against local Thai SIV viruses, A/swine/Thailand/CU-CB1/2006(H1N1) and A/swine/Thailand/CU-CB8.4/2007 (H3N2) viruses. The HI titers of pre-experiment and terminal sera of all experimental pigs against A/swine/Thailand/CU-RA29/2009 (H1N1) or pH1N1were not significantly elevated from the base line.

## Discussion

In this study, pigs in both pH1N1 and rH1N1infected cohorts showed typical SIV clinical signs such as sneezing and coughing from 1-4 DPI [17,20]. As expected, clinical signs of pigs inoculated with the pH1N1 virus and rH1N1 virus were unable to be distinguished from one another. It should be noted that viral isolation of nasal swabs from both infected groups demonstrated influenza A virus positivity until 6 DPI and only the real time RT-PCR showed positive results on 7-12 DPI suggesting that the duration of infectivity extended to 6 DPI. The modified RT-PCR used in this study appeared more sensitive than viral isolation. However, the infectivity from 7-12 DPI was inconclusive. The viral RNA could be detected as early as 1 DPI in both infected groups. But viral isolation results were only tested positive on 3-6 DPI in the rH1N1-infected group and 5-6 DPI in the pH1N1-infected group. Interestingly, the viral RNA was detected at 7-12 DPI in the pH1N1-infected pigs with mild concurrent clinical signs and histopathological lung lesions. Similar to the pH1N1-infected pigs, the rH1N1-infected pigs also showed prolong period of viral detection from the nasal swabs but in this cohort, the animals also had higher macroscopic lung lesions and the presence of virus antigen was noted in all sampled lung tissue.

Previous study on the pathogenesis of a Thai endemic SIV (H1N1) showed viral shedding between 2-4 DPI and a Thai endemic H3N2 had the shedding period only at 2 DPI [20]. Similarly, a study of human isolate pH1N1 in pigs demonstrated viral shedding as early as 1 DPI which persisted until the end of the experiment at 5 DPI [17]. In the present study, pigs in both infected groups showed detectable live viral shedding from 3-6 DPI based on viral isolation but the viral RNA was only sporadically detected through 12 DPI. The long shedding period may allow viral transmission among pigs as well as interspecies transmission particularly to the humans working in close proximity with infected pigs. In contrast to the rH1N1-infected pigs, there was no SIV antigen detected in the lung of the pH1N1-infected pigs. The sporadic viral detection in the lungs of the pH1N1-infected pigs possibly resulted from limited viral replication and fast viral antigen disappearing. Similar to the previous Thai endemic SIV-infected pigs, the studied viral RNA was detected in the respiratory tract of both infected groups and was not found in any other organ system [20]. In contrast to the study of pH1N1 (human origin) in pigs, viral RNA was also detected in tonsil, and serum [17].

Interestingly, the rH1N1-infected pigs demonstrated greater severity in term of clinical signs, pathological lesions and the overall number of pigs shedding the virus. As such, the reassortant virus theoretically could better infect pigs in comparison to the pH1N1. The only difference between the two studied viruses is the NA gene responsible for releasing the progeny viral particles from the infected cells [21]. Since rH1N1 obtained the NA gene from the local Thai SIV (97.2% amino acid sequence identity), the virus might be more compatible in Thai pigs when compared with the pH1N1 (99.6% amino acid sequence identity to human pH1N1 but 95% identity to other SIV isolates) [22]. However, the role of NA gene in SIV pathogenesis has not been fully elucidated and would require further investigation.

Importantly, the pH1N1 contains the triple reassortant internal gene (TRIG) cassette composed of swine, avian and human origin genes. It has been speculated that the TRIG cassette may be able to accommodate multiple HA and NA genes providing advantages to the viral infectivity, replication and possibly mutation. As a result, the TRIG cassette might be the cause of the reported increasing genetic variation rate of SIV in the US occurring since 1998 [9,23,24]. Since the TRIG cassette was recently introduced into the Thai pig population by the pH1N1 virus, the emergence of the Thai reassortant virus (rH1N1) in pigs has been described [12]. In addition, evidence of interspecies transmission among human and pig populations are occasionally reported [1,4,25]. Any novel rH1N1 influenza virus may be able to transmit back to the human population without being noticed and possibly causing another pandemic outbreak. As such, surveillance of influenza virus infections in both pigs and humans is critical for early recognition and prevention of a potential epidemic or pandemic outbreak.

## Conclusion

In summary, clinical manifestations and pathological lesions of both pH1N1 and rH1N1-infected pigs in this study were most evident during the early stages of infection (1-4 DPI), consistent with studies of the pathogenesis of other SIV infections. The rH1N1-infected pigs demonstrated prominent clinical signs and pathological lesions typical of SIV infection and nasal swab tests noted that the reassortant virus had higher numbers of pigs shedding the infective virus based on the viral isolation. While result is not statistically significant, the trend observed suggests both cohorts demonstrated some animals shedding virus through the end of the study at12 DPI. Similar to other SIV studies, the studied viruses replicated well in the lung tissues and the viral antigen was only detected within the respiratory tract.

## Materials and methods

### Viruses

A/swine/Thailand/CU-RA29/2009(H1N1) [7], a pandemic H1N1 of pig origin (pH1N1) and A/swine/Thailand/CU-SA43/2010 (H1N1) [12], a novel reassortant virus of pig origin (rH1N1) were individually propagated 3 times in 9-day-old embryonated chicken eggs. Allantoic fluids were collected after 72 hours incubation. The virus concentrations were calculated using 50% tissue culture infectious dose (TCID_50_) in Madin-Darby canine kidney (MDCK) cell using Reed and Muench method. Concentrations of both viruses were adjusted to 10^4^ TCID_50_/ml and kept in the -80°C until used.

### Experimental pigs

Twenty one, 3-week-old pigs from a local SIV, porcine circovirus type 2 (PCV2) and porcine reproductive and respiratory syndrome virus (PRRSV)-free herd (kindly provided by the Charoen Pokphand Food public company limited, Thailand) were divided into 3 groups. Group 1 and 2 containing 9 pigs each were intratracheally inoculated with 5 ml containing 10^4^ TCID_50_/ml of pH1N1 and rH1N1, respectively. A negative control group containing 3 pigs received mock cell culture media intratracheally. Clinical signs such as fever, coughing, sneezing, nasal discharge and conjunctivitis were blindly recorded daily by the same veterinarian for a week and at 10 and 12 days post infection (DPI). All pigs tested serologically negative for PRRSV and PCV2 using commercial ELISA kits (IDEXX laboratories, USA and Synbiotics, USA, respectively). All animals were housed in the animal facility biosafety level 2 with appropriated food and clean water providing adequately throughout the experiment. The animal usage and procedures were approved by Chulalongkorn University-Faculty of Veterinary Science animal care and use committee (protocol No. 11310052).

### Viral detection

Nasal swab were collected at 1-7, 10 and 12 DPI. Total RNA was extracted from nasal swabs, sera, fresh bronchial lymph node and lung tissue collected at necropsy by using a commercial kit (NucleoSpin Extract Viral RNA Kit, Macherey-Nagel, Germany). A modified real time reverse transcriptase polymerase chain reaction (real time RT-PCR) was performed using Superscript III platinum one-step quantitative RT-PCR system (Invitrogen, USA). Primers specific to Matrix (M) gene containingforward primer (MF3; 5’ TGATCTTCTTGAAAATTTGCAG 3’), reward primer (MR1+; 5’ CCGTAGMAGGCCCTCTTTTCA 3’) and M-probe (FAM-TTGTGGATTCTTGATCG-MGB) were used in this study. The cycling conditions started at 48°C for 45 min, 95°C for 10 min and followed by 40 cycles of denaturation (94°C for 15 s), annealing (55°C for 30 s) and extension (72°C for 40 s) [26].

Nasal swabs, lung and bronchial lymph node homogenate samples were filtrated and inoculated onto MDCK cells using ten-fold serial dilutions. The inoculated cell cultures were incubated for 72 hours. Virus was identified using anti-influenza A nucleoprotein monoclonal antibody as a primary antibody and rabbit anti-mouse IgG conjugated horseradish peroxidase as a secondary antibody (DakoCytomation, Carpinteria, California). Then, color was developed using a chromogen aminoethylcarbazole substrate (Sigma, St. Louis, Missouri) [20].

### Pathological examination

Three pigs from each viral inoculated group and 1 pig from the negative control group were randomly selected for euthanasia and necropsied at 2, 4 and 12 DPI. At necropsy, percentages of gross lung lesion scores characterized by multifocal mottled tan and consolidation in consistency were recorded and scored as previously described [20]. Lung, bronchial lymph nodes, ileum, tonsil, liver, kidney and spleen were collected from each animal at necropsy, immersed and fixed in 10% buffered formalin for subsequent histopathological analysis.

Formalin-fixed tissues were embedded in paraffin and processed routinely. Sections were cut approximately 4-6 μm thick for histopathological and immunohistochemistry (IHC) staining for Influenza A virus antigen detection. The IHC staining was performed using a labeled streptavidin-biotin (LSAB) method. Primary antibody using anti-influenza A (H5N1) nucleoprotein monoclonal mouse antibodies (EVS238, B.V.EUROPEAN VETERINARY LABORATORY, the Netherlands) and secondary antibody using Biotinylated rabbit anti-mouse IgG antibody and envision polymer (Envision Polymer DAKO®, Denmark) were concurrently performed with a negative control slide. The sections were developed with 3, 3’-diaminobenzidine tetrahydrochloride (DAB) and counterstained with Mayer’s hematoxylin. A positive control slide was also included using the SIV-infected lung section from our previous experiment [7].

### Hemagglutinination inhibition (HI) assay

Sera were collected from all pigs before starting the experiment and at each necropsy. All sera were pretreated with 20% kaolin and receptor destroying enzyme (Denka Seiken Co. Ltd., Japan). The antibody detection was performed used standard HI assay [1]. Virus antigens used in this experiment were representatives of Thai endemic swine viruses; A/swine/Thailand/CU-CB1/2006(H1N1) and A/swine/Thailand/CU-CB8.4/2007 (H3N2) and pH1N1 virus (A/swine/Thailand/CU-RA29/2009(H1N1)). Samples with HI titers ≥ 40 were considered as previously exposed to the specific tested antigen.

## Abbreviations

pH1N1: Pandemic H1N1 2009 virus; rH1N1: Pandemic H1N1 2009 reassortant virus; DPI: Days post infection; SA: Sialic acid; SIV: Swine influenza virus; TRIG: Triple reassortment internal gene cassette; M: Matrix gene; NA: Neuraminidase gene; HA: Hemagglutinin gene; PB2: Polymerase basic 2 gene; PB1: Polymerase basic 1 gene; PA: Polymerase acidic gene; NP: Nucleoprotein gene; NS: Non-structural protein gene; RT-PCR: Reverse transcriptase – polymerase chain reaction; IHC: Immunohistochemistry staining; HI: Hemagglutination-inhibition test; RNA: Ribonucleic acid; TCID50: 50% Tissue culture infective dose; MDCK: Madin-darby canine kidney cell line, ml, milliliter; C: Degree celsius; PCV2: Porcine circovirus type 2; PRRSV: Porcine reproductive and respiratory syndrome virus; ELISA: Enzyme-linked immunosorbent assay; s: Second; LSAB: Labeled streptavidin-biotin method; DAB: 3, 3’-diaminobenzidine tetrahydrochloride.

## Competing interests

The authors declare that they have no competing interests.

## Authors’ contributions

NC carried out virology, pathology, molecular genetic study and animal experiment, analysis the data and drafting the manuscript, JK carried out animal work, virology and molecular genetic study, DS carried out animal work and virology, ST, SJ, JA carried out animal work, AA carried out molecular genetic study, RT carried out experimental design, pathology, and drafting and revising the manuscript. All authors have read and approved the final manuscript.
